# The *in vivo* blood compatibility of bio-inspired small diameter vascular graft: effect of submicron longitudinally aligned topography

**DOI:** 10.1186/1471-2261-13-79

**Published:** 2013-10-01

**Authors:** Ruiming Liu, Yuansen Qin, Huijin Wang, Yong Zhao, Zuojun Hu, Shenming Wang

**Affiliations:** 1 Department of Vascular Surgery, First Affiliated Hospital of Sun Yat-sen University, Guangzhou 510080, P. R. China; 2 Key Laboratory of Bio-Inspired Smart Interfacial Science and Technology of Ministry of Education, School of Chemistry and Environment, Beihang University, Beijing 100191, P. R. China

**Keywords:** Vascular grafts, Electrospinning, Aligned topography, Thrombosis, Platelet adhesion

## Abstract

**Background:**

Cardiovascular disease is the leading cause of deaths worldwide and the arterial reconstructive surgery remains the treatment of choice. Although large diameter vascular grafts have been widely used in clinical practices, there is an urgent need to develop a small diameter vascular graft with enhanced blood compatibility. Herein, we fabricated a small diameter vascular graft with submicron longitudinally aligned topography, which mimicked the tunica intima of the native arterial vessels and were tested in Sprague–Dawley (SD) rats.

**Methods:**

Vascular grafts with aligned and smooth topography were prepared by electrospinning and were connected to the abdominal aorta of the SD rats to evaluate their blood compatibility. Graft patency and platelet adhesion were evaluated by color Doppler ultrasound and immunofluorescence respectively.

**Results:**

We observed a significant higher patency rate (*p* = 0.021) and less thrombus formation in vascular graft with aligned topography than vascular graft with smooth topography. However, no significant difference between the adhesion rates on both vascular grafts (smooth/aligned: 0.35‰/0.12‰, *p* > 0.05) was observed. Moreover, both vascular grafts had few adherent activated platelets on the luminal surface.

**Conclusion:**

Bionic vascular graft showed enhanced blood compatibility due to the effect of surface topography. Therefore, it has considerable potential for using in clinical application.

## Background

Cardiovascular diseases are the leading cause of morbidity and mortality worldwide, and arterial reconstructive surgery of the heart or the lower extremity remains the common surgical solution for a number of patients [1]. In the United States alone, about 1.4 million cases of arterial bypass operations are performed each year [2]. Despite the clinical success of large diameter (>6 mm) vascular graft [3], the patency rates of small diameter (<6 mm) vascular graft are very poor [4-7], which largely limit their application in coronary and peripheral vascular bypass graft procedures. As thrombosis at the blood-material interface is the predominant cause of the failure of small diameter vascular graft [8], it is necessary to develop a new type of small diameter vascular graft with enhanced blood compatibility [9,10].

Polyurethane (PU), which has excellent antithrombogenicity, biocompatibility and elastic mechanical properties, has become the principal candidate material for small diameter vascular grafts [11-14]. However, as far as its blood compatibility is concerned, PU is still far from the ideal. As an ideal blood compatible material, after contacting with blood, it doesn’t damage blood components and/or change the structure or function of plasma proteins [15]. From the material science point of view, a native blood vessel is the best blood compatible material. The intimal layer of blood vessel can resist platelet adhesion and prevent undesirable thrombus formation [16]. It has been found that the tunica intima of abdominal aorta in rat was composed of submicron-scaled grooves and ridges along the longitudinal axis and nano-scaled protuberances. Thereafter, a planar polydimethylsiloxane-based material with multiscale interlaced submicron ridges and nano-protrusions was developed [17]. This biomimetic topography can effectively reduce the adhesion of activated platelets under flow conditions. However, such planar material with bionic micro/nano structure can’t be used in vascular bypass surgery without 3D vascular graft configuration.

Electrospinning is a very effective method to fabricate nanofiber-based tissue engineering scaffolds which bear biomimetic extra cellular matrix topography and considerable mechanical characteristics [18,19]. Most nanofibers produced by electrospinning have a random microstructure, however, many human tissues have anisotropic microstructures such as blood vessel, nerve conduits and ligaments [20-22]. Several researches have proved vascular graft with circumferentially aligned topography, which mimics the anisotropic microstructures of the media layer in native blood vessel, can regulate macroscopic mechanical properties and guide the regeneration of vascular tissue [23,24]. So far, there are few researches about the fabrication of vascular graft with longitudinally aligned topography and its influence on blood compatibility.

In the present study, a bionic small diameter vascular graft with longitudinally aligned topography was fabricated. The vascular grafts were connected to the abdominal aorta to assess the platelet adhesion, thrombosis and patency rate, with vascular graft bearing smooth topography as a control. The results indicated the bionic vascular graft had improved blood compatibility.

## Methods

### Preparation and characterization of vascular grafts

Two types of vascular grafts i.e. vascular graft with aligned topography and vascular graft with smooth topography were prepared as follows;

#### Vascular graft with aligned topography

**Figure 1:**
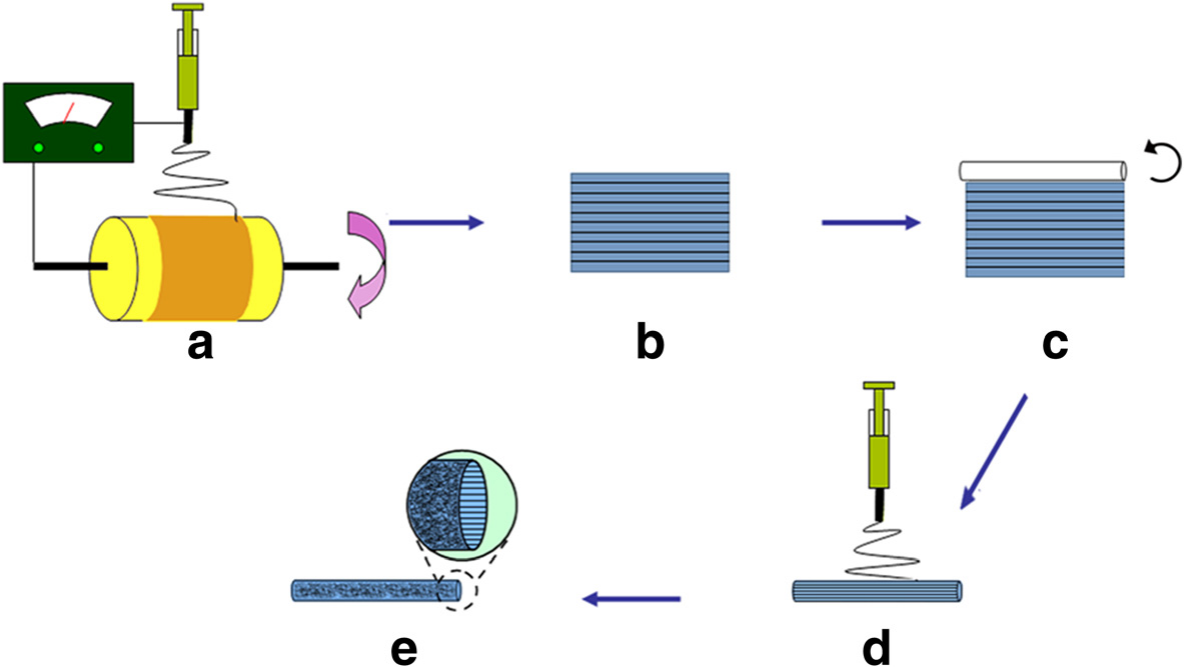
**Schematics of fabricating vascular graft with submicron longitudinally aligned topography. (a)** Electrospinning PU onto high velocity rotating drum. **(b)** Removing the aligned nanofibrous mat from the drum. **(c)** Production of tubular construct by rolling the nanofibrous mat around a metal needle. **(d)** Electrospinning of random PU nanofibers on the top of aligned tubular scaffold. **(e)** The vascular graft with aligned intimal topography and random external surface.

This graft was prepared by dissolving a medical graded polyurethane (PU) (Sigma-Aldrich, Hong Kong, China) in Tetrahydrofuran (THF)/N, N-Dimethylformamide (DMF) (Yili Fine Chemical Co., Ltd., Beijing, China) (3:7, w/w) to make 13% PU solution. The schematics of fabricating vascular graft with biomimetic topography are represented in Figure 1. The solution was fed through a syringe pump at a flow rate of 0.6 mL h^-1^. A rotating drum covered with aluminum foil acted as the collector. The distance between the collector and the tip (23-G) was 25 cm, and the applied voltage to the tip was 25 kV. An aligned nanofibrous mat was fabricated at a rotating speed of 5000 rpm for 0.5 h. Thereafter, the aligned nanofibrous mat was rolled around a metal needle (1 mm) to produce a tubular form, which acted as the intimal layer of vascular graft. Then the vascular graft with aligned topography was thickened by electrospinning PU solution onto the as-prepared tubular construct at a rotating speed of 500 rpm. The condition of electrospinning was the same as aforementioned, and 3 mL solution was used for each graft.

#### Vascular graft with smooth topography

To produce the vascular grafts with smooth topography, a smooth film was prepared from the same PU solution described above by spin coating on a silicon wafer. Prior to spin coating, silicon wafer was cleaned by rinsing with acetone, methanol and isopropanol several times and dried under nitrogen. The PU solution was then spin coated onto the silicon wafer at 1000 rpm for 25 s. The construction of intimal layer with smooth mat and external layer with random PU nanofibers was the same as provided above.

The vascular grafts samples were sputter coated with gold, and characterized by field-emission scanning electron microscopy (JEOL JSM-6700 F). NIH ImageJ software was used to analyze the electrospinning fibers size.

### Cannula insertion

Fourteen Adult Sprague–Dawley rats (WeiTongLiHua animal center, Beijing, China), weighing 400 ± 20 g, were randomly divided into two groups: cannula insertion with vascular graft bearing aligned topography (n = 7) and smooth topography (n = 7). Each rat was operated in the supine position under general anesthesia with pentobarbital sodium (50 mg/kg ip), and approximately 1.2 cm length abdominal aorta was exposed using blunt and sharp dissection. After clamping the distal and proximal portion of the abdominal aorta, the vascular graft (1 cm in length), which had been previously connected to cannulas (outer diameter 1.5 mm) on both ends, was inserted into the abdominal aorta. Then the bull-dog clamps at the distal and proximal end were removed sequentially to restore the blood flow. All surgical protocols were approved by the Institutional Animal Care and Use Committee at the First Affiliated Hospital of Sun Yat-sen University (Permit Number: 2012–019), and every effort was made to minimize suffering.

### Measurement of Graft patency and platelet adhesion

The patency (the state of being open or unobstructed) of the vascular grafts can be initially evaluated by palpation of the distal artery, and then confirmed by the color Doppler ultrasound (HDI 5000, Philips Medical Systems, Bothell, WA, USA) at 15 min. After that, the vascular grafts were harvested, opened longitudinally and photographed. The removed grafts were flushed with PBS to remove the residual blood, and horizontally divided into two pieces. One piece was fixed with 2.5% glutaraldehyde at 4°C for 4 hours, and then dehydrated with a series of ethanol solution (30, 50, 70, 85, 95 and 100%), followed by critical point dried, finally observed by a low vacuum scanning electron microscope (SEM) (FEI Quanta 200). The other piece was fixed in 95% ethanol for 20 min, and then indirect immunofluorescence was performed to measure quantitatively platelet adhesion. The anti-CD62P mAb (Abcam, Cambridge, UK) was used as the primary antibody [25] and FITC-labeled goat anti-mouse antibody (Abcam, Cambridge, UK) as the secondary antibody. After immunofluorescence staining, the graft was observed with fluorescence microscopy (Nikon Ti-E) equipped with DG-4 (Sutter Instrument), and analyzed with Metamorph software.

### Statistical analysis

The adhesion rates were expressed as the median and the other results were shown as mean ± SD. Statistical tests were performed using SPSS for Windows. Fisher’s exact probabilities were used to assess differences in patency rates and non-parametric test was applied to compare the platelet adhesion rate of two groups. The *p* values less than 0.05 were considered to be significant.

## Results

### Morphological assessments

**Figure 2:**
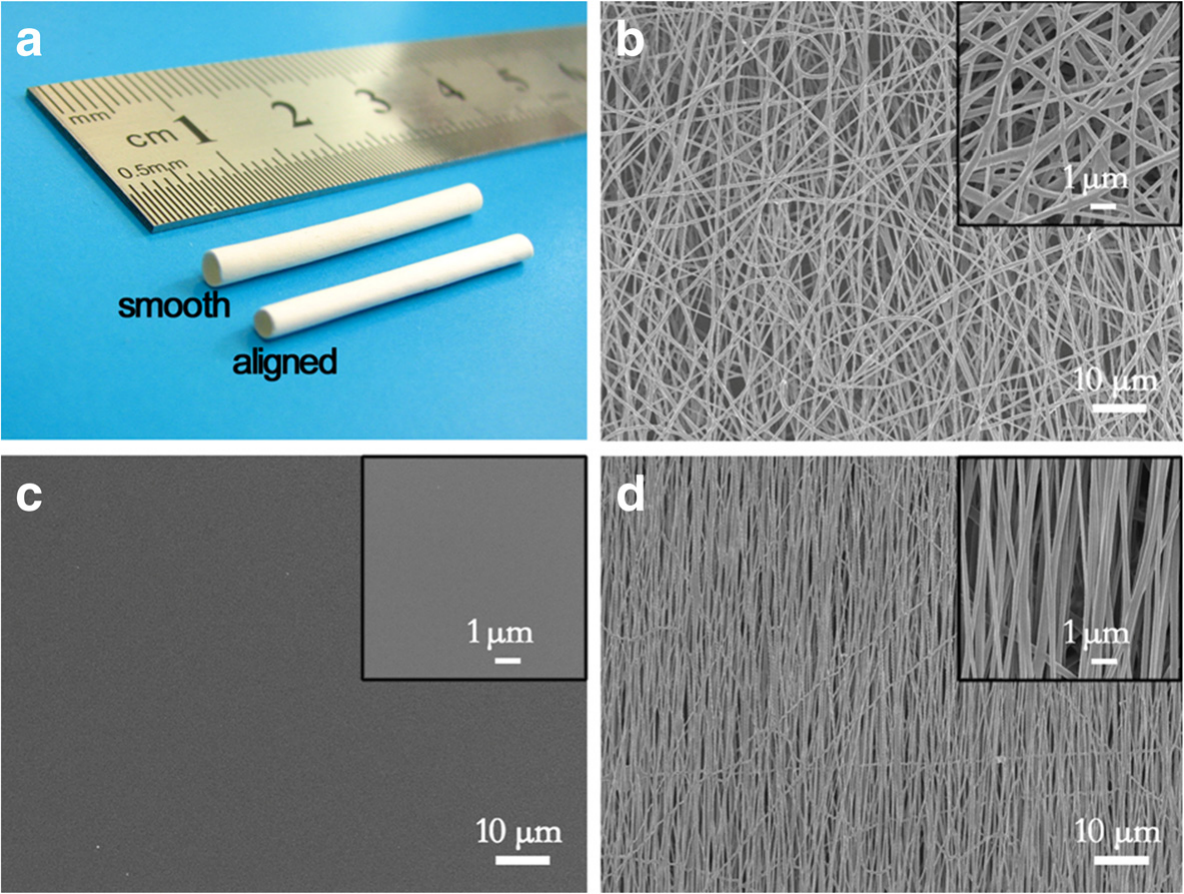
**Morphology of vascular grafts. (a)** Macroscopic image of vascular grafts with different topography. **(b)** SEM image of external surface topography on vascular grafts. **(c)** ~ **(d)** SEM image of smooth, aligned topography on vascular grafts. The vascular grafts had similar external surface topography, while different intimal topography.

Both types of the vascular grafts i.e. vascular graft with smooth topography and vascular graft with aligned topography had smooth and flawless appearances (Figure 2a). The inner diameter of the vascular graft was 1.5 mm, and the wall thickness was 210 ± 30 μm. The SEM image showed the external surface of the vascular grafts were both composed of random nanofiber networks (Figure 2b), while the intimal surface of them had different topography, namely smooth topography (Figure 2c) and aligned topography (Figure 2d). The diameters of nanofiber in the external and intimal surface were 350 ± 80 nm and 267 ± 40 nm respectively.

### The patency rate of vascular grafts and thrombosis

**Figure 3:**
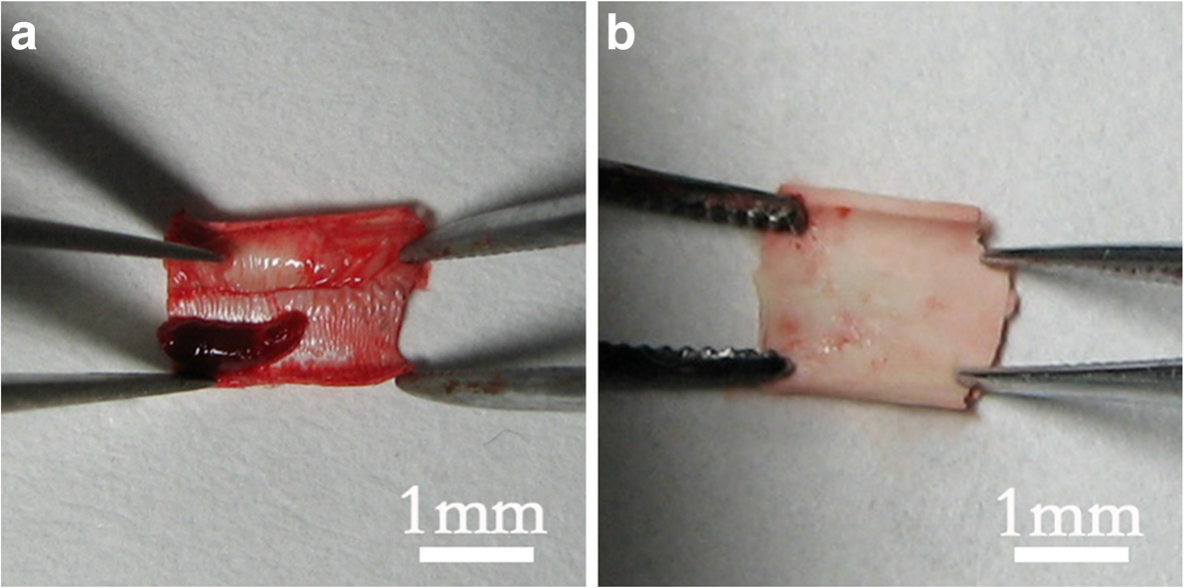
**Thrombosis on vascular grafts. (a)** Obvious thrombus formation on the vascular graft with smooth topography. **(b)** Less thrombus formation on the grafts with aligned topography.

The patency rates of vascular grafts were listed in Table 1. The vascular graft with aligned topography had significantly higher (Table 2) patency rate than vascular graft with smooth topography. After removing from the abdominal aorta, there was much less thrombus formation on vascular grafts with aligned topography (Figure 3) as compared to the vascular graft with smooth topography. These results were consistent with the result of patency rates.

**Table 1:** **Fisher’s exact probabilities for comparison of patency rate of vascular grafts with different topography (*p*** **= 0.021)**

***Intimal topography***	***Vascular occlusion***	***Vascular patency***	***Total***	***Patency rate***
*Smooth topography*	*5 (2.5)*	*2 (4.5)*	*7*	*28.60%*
*Aligned topography*	*0 (2.5)*	*7 (4.5)*	*7*	*100%*
*Total*	*5*	*9*	*14*	*-*

The number in the bracket represents expected count. As N < 40, Fisher’s exact probability was chosen to compare the patency rate of vascular grafts with different topography.

**Table 2:** Non-parametric test result for comparing platelet adhesion rate of vascular grafts with different topography

***Adhesion rate-s***^***†***^	***Adhesion rate-a***^***‡***^	***Test statistic***	***p***
*0.35‰*	*0.12‰*	*Mann–Whitney Test*	*0.058*

^**†**^ Adhesion Rate-S stands for adhesion rate of vascular graft with smooth topography.

^**‡**^ Adhesion Rate-A stands for adhesion rate of vascular graft with aligned topography.

### *In vivo* platelets adhesion

**Figure 4:**
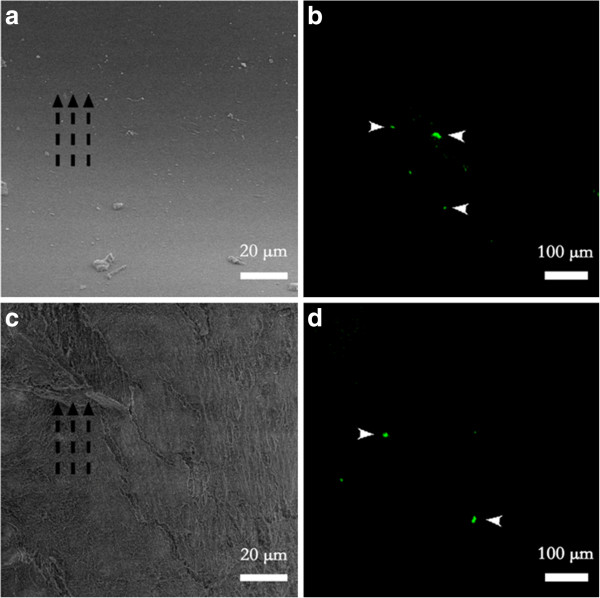
**Low vacuum SEM and immunofluorescent image of platelet adhesion on vascular grafts with different topography. (a)** Nothing but small amount of cellular debris adhered on the smooth topography. **(c)** Protein-like substances adhered on the aligned topography along the direction of blood flow and aligned nanofibers. **(b)** ~ **(d)** Few platelets adhered on both topography. Broken black arrows indicate the direction of the blood flow, and white arrows indicate the adhered platelets.

The low vacuum SEM image showed that there were few platelet clots adhered on the both vascular grafts (Figure 4a and 4c). However the luminal surface conditions were different, that is, the luminal surface of vascular grafts with smooth topography was almost bare except a small amount of cellular debris, whereas the surfaces of aligned ones were covered by protein-like substances.

Platelet adhesion was also quantified with immunofluorescence experiments. Both of the vascular grafts had few activated platelet adhesions on the luminal surface (Figure 4b and 4d), which was in accordance with our results of SEM. With the help of software Metamorph, we calculated the adhesion rate of the activated platelets on different vascular grafts using the following equation:M

$$\begin{array}{l} \mathrm{Adhesion}\;\mathrm{rate}‰=\frac{\mathrm{area}\;\mathrm{adhered}\;\mathrm{with}\;\mathrm{activated}\;\mathrm{platelets}}{\mathrm{total}\;\mathrm{field}\;\mathrm{area}}\\ \;\times 1000‰ \end{array}$$

We observed that there was no significant difference between the adhesion rates on different vascular grafts (smooth/aligned: 0.35‰/0.12‰, *p* = 0.058), although they had showed an obvious difference in patency rate (Table 2). Though two types of vascular grafts had different luminal surface conditions, they both showed little platelet adhesion. Considering that the vascular graft with smooth intimal topography had lower patency rates and more thrombus, there may be different reasons for less platelet adhesion.

## Discussion

After 4 billion years of evolution, nature has developed a wide variety of amazing structures and functions [26], therefore learning from nature can pave the way for designing and preparing new materials. It has been proved in most cases that the structure especially micro/nano structure of the natural biomaterials often determine its function in the actual situation [27-30]. The new type of vascular graft we designed has similar submicron longitudinally aligned topography with the native blood vessel, and it also showed higher patency rate and less thrombus formation.

The adhesion of platelets to blood-contacting surface directly influenced the blood compatibility of vascular graft. Once platelets adhered, they become activated and aggregate to form platelet thrombus, and then gradually diminish blood flow, consequently lead to vascular occlusion [31]. A commonly accepted fact is that increase the surface roughness can lead to more platelet adhesion, because extra surface roughness usually means larger area exposed to the platelets [32]. However, with the development of nanotechnology, the definition of roughness is further refined on the basis of roughness dimensions, and the roughness dimensions can impact the relationship between roughness and platelet adhesion. It has been proved that the materials with micrometer-scale topography exhibit more platelet adhesion at early blood contact times (2 to 5 min) [33,34]; however, the ones with submicron-scale topography can decrease the number of the adhered platelets [35,36]. In our study, we chose the vascular graft with smooth topography as the less surface roughness model, and evaluated the influence of surface topography on the blood compatibility through the platelet adhesion, patency rates and thrombosis. As the dimension of topography on the bionic vascular graft (267 ± 40 nm) is smaller than the diameter of individual platelet (2–4 μm), the effective contact area between platelets and actual surface is reduced, which decreases the number of the adhered platelets.

Under flow conditions, the collision frequency of platelets with the surface can also influence the interaction between material surface and platelets, which is negatively correlated with the velocity of the boundary layer [37,38]. Previous research has indicated that if a hydrophobic surface is covered with micro/nanostructure such as posts, grooves, or others, the interstitial pores of patterned structure will not be filled with liquid due to the effect of surface tension. This non-wetting state will reduce solid/liquid contact area and friction of the fluid passing the boundaries, resulting in increased boundary velocity [39-41]. In our study, the PU vascular graft with biomimetic topography had a hydrophobic surface (contact angle 132.5 ± 3.5°) and submicron longitudinally groove-like topography. They concertedly remodeled the boundary conditions of the blood flow, which in turn largely accelerated the velocity of the boundary layer and decreased the collision frequencies of platelets with the surface, consequently leading to less platelet adhesion.

It is interesting that vascular grafts with smooth topography also had few activated platelets adhered on the surface, but both the lower patency rate and more thrombosis indicated that there were different reasons for that. We speculated that when blood flowed through the graft, the platelets were activated, aggregated and thrombus formation was triggered. Then the thrombus was detached from the grafts by the blood flow of abdominal aorta, and embolized the distal portion of the vessels. Actually, the *in vitro* platelet adhesion experiments under flow condition had confirmed that the vascular graft with smooth topography had more platelet adhesion in highly activated form [42]. Our results, once again, indicated the importance of minor differences in the surface topography in directing a desired platelet response.

## Conclusions

In summary, we fabricated a small diameter vascular graft with submicron longitudinally aligned topography by electrospinning technique, and evaluated the blood compatibility. The results indicated that the bionic vascular graft showed enhanced blood compatibility due to the effect of surface topography, and could be very promising in clinical applications.

As platelet adhesion, which we focus on in this study, occurred shortly after restoring the blood flow, and acute occlusive thrombosis of rat can happen within 15 min [43-45], we choose 15 min as a time point to evaluate the hemocompatibility of vascular grafts. However, further *in vivo* studies such as long term implantation are needed to confirm our findings.

## Competing interests

The authors declare that there is no potential or relevant competing financial or other interest that might be affected by publication of the results contained in the manuscript.

## Authors’ contributions

RM L carried out the preparation and characterization of vascular grafts, immunofluorescence and drafted the manuscript. YS Q and HJ W carried out the animal preparation and cannula insertion; they also contributed to statistical analysis and interpretation of data. ZJ H, SM W and Y Z designed, set up and monitored the study. All authors read and approved the final manuscript.

## Pre-publication history

The pre-publication history for this paper can be accessed here:

http://www.biomedcentral.com/1471-2261/13/79/prepub
